# Cannabis, Schizophrenia Risk and Genetics: A Case Report of a Patient With Homozygous Valine Catechol-O-Methyltransferase Polymorphism

**DOI:** 10.7759/cureus.15740

**Published:** 2021-06-18

**Authors:** Katelyn Grechuk, Heela Azizi, Vatsala Sharma, Tasmia Khan, Ayodeji Jolayemi

**Affiliations:** 1 Psychiatry, Saba University School of Medicine, Saba, BES; 2 Psychiatry, American University of Antigua College of Medicine, New York, USA; 3 Psychiatry, Interfaith Medical Center, New York, USA; 4 Psychiatry, Medical University of the Americas, New York, USA

**Keywords:** cannabis, schizophrenia, comt, gene polymorphisms, psychosis, val158met, genetics

## Abstract

The question of whether cannabis can trigger schizophrenia continues to be a subject of interest. There has been an increasing focus on identifying potential genetic factors that may predispose cannabis users to develop schizophrenia. One such gene identified in many studies codes for a catechol-O-methyltransferase (COMT) enzyme polymorphism. These studies, however, are limited by the inclusion of patients displaying psychotic symptoms during cannabis intoxication and those who continue to display psychotic symptoms after its cessation. The latter is of interest in truly understanding the risk of cannabis triggering schizophrenia and more studies are needed to clarify the potential relationship. We present the case of a 24-year-old female who presented with psychotic symptoms and was diagnosed with schizophrenia after extensive cannabis use. In addition, she had a homozygous valine COMT polymorphism, a genetic variant thought to be associated with a predisposition for schizophrenia in cannabis users. We discuss the significance of our findings in understanding the relationship between cannabis use and the development of schizophrenia in genetically predisposed individuals.

## Introduction

Schizophrenia is a disorder that affects nearly 1% of the population worldwide [1]. In 2016, it was estimated that 3.9% of people around the world between the age of 15 and 64 were using cannabis, a 16% increase since 2006 [2]. There has been concern that cannabis use may cause schizophrenia in some individuals, and with such increasing use, a substantial amount of research has been focused on this hypothesis. 

Early retrospective studies were overall inconclusive. It was difficult to differentiate those who may have developed schizophrenia as a sequela of cannabis use from subjects with a predisposition who may have developed schizophrenia anyway, or previously apparent symptoms of schizophrenia that could have made one more likely to use cannabis. More recent, well-controlled studies have concluded that cannabis use probably is a risk factor for schizophrenia in certain individuals, especially when used in adolescence and/or young adulthood [3, 4]. 

Schizophrenia is a multifactorial disorder with a high heritability rate [1]. Thus, it is likely that both environment and genetics play a significant role in its development. The latest research on understanding schizophrenia has focused on finding a potential correlation between cannabis use and schizophrenia risk in those with a genetic predisposition [1]. One of the genes of interest is on chromosome 22 and codes for the enzyme catechol-O-methyltransferase (COMT), which is believed to interact with cannabis [1]. 

Exposure to cannabis activates D2 receptors and increases dopaminergic transmission in the striatum and mesolimbic areas of the brain.The prolonged exposure to cannabis leads to reduced dopamine in the prefrontal cortex [5]. The COMT gene encodes an enzyme responsible for degrading most of the prefrontal cortical dopamine. The COMT Val158Met functional polymorphism regulates enzymatic activity. The val allele results in increased prefrontal dopamine catabolism and therefore, lower extracellular dopamine level [5]. It further increases the risk of psychosis by depleting prefrontal dopamine availability and increasing mesolimbic dopaminergic activity in the feedback loop [5]. There is limited evidence for the mechanism of direct cannabis - COMT interaction in schizophrenia susceptibility [6].

To further explore the relationship between cannabis use and schizophrenia risk in genetically susceptible individuals, we present a case of a 24-year-old female with a history of chronic cannabis use and a homozygous valine COMT polymorphism. After years of consistent cannabis use throughout her adolescence and early adulthood, she developed psychotic symptoms and was diagnosed with schizophreniform disorder. Therefore, we explore the implications of our findings in light of the research previously reported.

## Case presentation

A 24-year-old Hispanic female was brought to the Emergency Department (ED) by her grandparents with the chief complaint of increasingly depressed mood, bizarre behavior and thought content, and multiple delusions for the past two months. Her grandparents contacted emergency services when the patient claimed that “aliens were taking her blood.” The patient exhibited reduced sleep, decreased appetite and poor self-care that began four months prior and continuously escalated. She was notably disheveled and malnourished. Her speech was slow and soft with exaggerated latency and decreased intonation. There were no physical signs of drug abuse. Her behavior was generally reclusive. Eye contact was poor at times and excessive at others. She was uncooperative and displayed unprovoked, explosive hostility. Thought process displayed ruminative ideas and paranoid, persecutory and bizarre delusions without hallucinations. Her affect was mostly low, but labile and congruent to her stated mood. The patient displayed poverty of speech and psychomotor retardation. She was uncooperative with cognitive testing pertaining to memory, orientation, and attention. The patient reported anhedonia, significant guilt and decreased appetite as a sequelae of her delusions. She denied suicidal ideation or changes in energy, and concentration. She displayed poor judgement and limited insight into her illness. 

The patient was admitted to the psychiatric unit where she endorsed visions “upon which she could not elaborate and found too powerful to explain.” Her grandparents reported a four month “obsession with pyramids and aliens'' and gradual, bizarre behavioral changes. The patient expressed immense and inexplicable guilt for “hurting her family and everyone else.” She attributed these feelings and the “pain she had caused” to her cannabis use. The patient began smoking cannabis recreationally at age 13, at which time she would share “a blunt” with friends about once a week. Her cannabis use continued steadily until the age of 17, when it increased dramatically. From age 17-24 (age of presentation), she reported using cannabis daily. By 24 years of age, she reported smoking five to six joints per day. She reported quitting four months prior to admission because she was experiencing new-onset persecutory feelings and hypervigilant behavior that she attributed to her cannabis use. She denied any other drug use, but reported smoking cigarettes more frequently during this time due to life stressors and cessation of cannabis use. She had been smoking about a half pack of cigarettes a day for the past four months and was abstaining from alcohol. She denied the use of any other substance. With the onset of her symptoms four months prior to admission, the patient resigned from her job. Of note, one month prior to her presentation, the patient suddenly lost her long-term partner and her condition further deteriorated. Her family reported progressively worsening mood, increased reclusiveness, and bizarre thought content and behavior throughout this time. 

The patient’s premorbid functioning was optimal. She excelled in school and extracurricular activities, was well-liked by her peers, and had many friends. Prior to symptom onset, the patient worked as a licensed real estate agent and lived with her grandparents and father. Pertinent family history is significant for schizophrenia in her father. The patient had no known significant past psychiatric or medical history and was taking no medications prescribed or otherwise.

**Figure 1:**
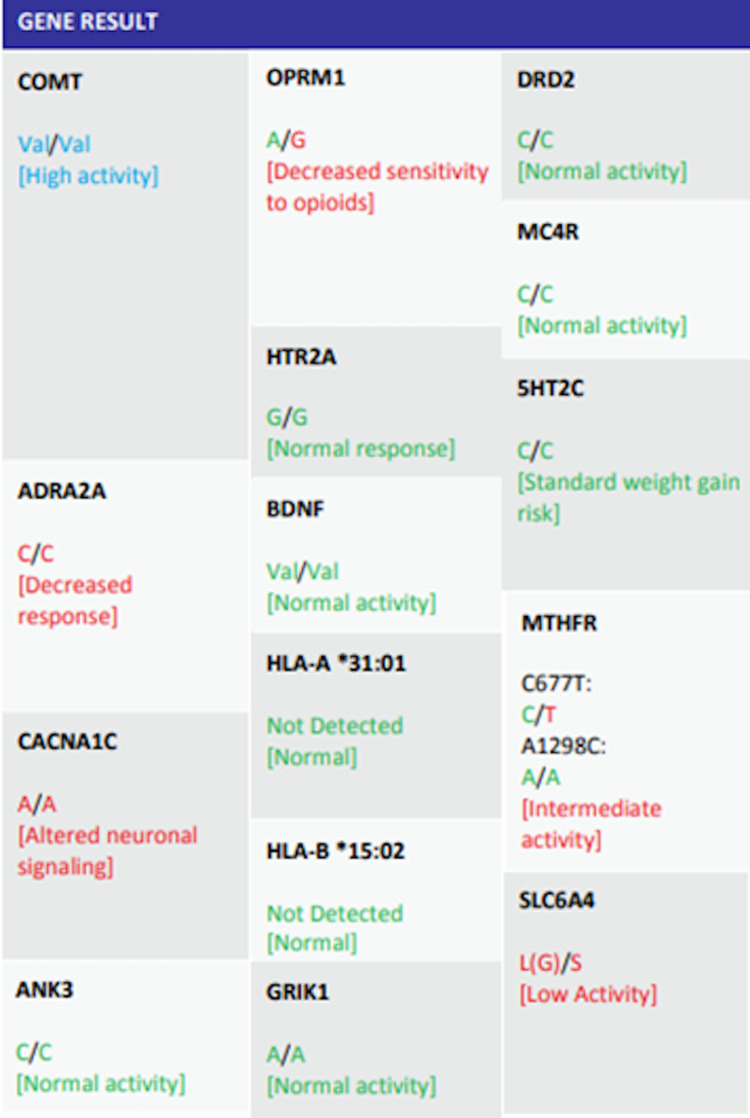
GeneticTest Results For the Patient

At the time of admission, all parameters of physical examination and lab results were within normal limits. Non-contrast CT of the head was unremarkable. Urine toxicology report was negative for all substances. Complete blood count and urinalysis were normal. A pharmacogenetic profile test revealed homozygous valine COMT polymorphism. The procedure involves obtaining an oral swab to sample DNA and then performing an assay using real-time PCR. Specific gene variants that are linked with particular patient characteristics are detected. Results for the patient are shown in Figure 1.

Upon admission, the patient was treated with risperidone 1 mg twice daily, sodium valproate 500 mg twice daily, and nicotine gum as needed. She initially refused medications and was belligerent and disruptive on the unit. Psychotic symptoms appeared to be exacerbated by visits from her grandparents. Thus, visitation restrictions were applied for several days. After psychoeducation on the importance of medication compliance, she agreed to take mood stabilizing and antipsychotic medications several times, as well as a one-time injection of paliperidone palmitate 234 mg IM one week post-admission. It was well tolerated and she did not develop any adverse effects. Evaluation on day eleven of admission revealed improvement in both psychotic and mood symptoms; the patient was less disruptive and grooming and hygiene had improved. A week after the initial dose, she received a second dose of paliperidone palmitate 156 mg IM on day 13 of admission and again, it was well tolerated. Her serum valproate concentration was found to be within the optimal range at 50 ug/mL. Her mood continued to stabilize, she became pleasant, agreeable, and cooperative; thought content normalized. She began participating in group activities and was interacting appropriately with others; no behavior issues were reported. Her impulse control improved and paranoia and delusions remitted; she was able to understand that previously endorsed psychotic beliefs and delusions were surreal. She became free of prior hostility and was able to engage in meaningful conversations. Visitation restrictions were lifted and her grandparents reported this behavior as her baseline. Fifteen days post-admission, the patient was discharged. At the time of discharge, she appeared calm, cooperative, and described her mood as “happy.” Her affect was full, appropriate, and reactive. Her thoughts were linear, logical, and organized. She denied any suicidal or homicidal ideation and appeared to be safe to herself and others. She was discharged on sodium valproate 500 mg twice daily and nicotine gum, as well as a plan for follow-up to continue paliperidone palmitate 156mg IM every 28 days. She had plans for the future that included regaining employment, travel with her family, continued compliance with medication and pursuing psychotherapy on an outpatient basis. A PANSS score was obtained retrospectively for when she arrived and compared to a score obtained when symptoms resolved. Her comparative scores upon admission (P=41, N=33, G=83), and discharge (P=7, N=7, G=26) depicted significant improvement in symptomatology and achievement of baseline functioning [1]. 

A week following discharge, the patient developed acute dystonia. She presented to the ED with a one-day complaint of dry mouth with numbness and heaviness of the tongue and slurred speech. She was treated with a single dose of benztropine 2 mg IM. Upon re-evaluation one-hour post-injection, the patient reported resolution of the presenting complaints. Two hours later, she was discharged from the ED with the addition of benztropine 2 mg PO once a day to her treatment regime. The patient was managed on an outpatient basis thereafter.

## Discussion

The patient presented without hallucinations, but with significant paranoid, persecutory and bizarre delusions. A medical work up was unrevealing of any metabolic, infectious, or neurological causes of psychosis. This patient met the DSM-5 criteria for the diagnosis of schizophreniform disorder, with symptoms of delusions, disorganized speech and behavior, and numerous negative symptoms. Her symptoms were present for four months prior to treatment with no previous episodes and no recurrence of psychosis or other symptoms. The negative symptoms she experienced included anhedonia, alogia, avolition and affective and relational deficits, but she did not meet the criteria for major depressive disorder. Her poor insight and lack of awareness of her illness further suggests these symptoms are negative symptoms of schizophrenia rather than depression. On further exploring the nature of these symptoms, it is noteworthy that these negative symptoms were secondary to schizophrenia; the patient reported that she started experiencing these negative symptoms with the onset of her delusions. She was treatment-naive and had discontinued cannabis use four months prior to their onset, which correlated with the onset of her psychotic symptoms. Therefore, the major indicative factor of her negative symptoms was their appearance secondary to the delusions. They were not related to any medication or substance use in this case. 

Two findings of note in this case were an extensive history of cannabis use and genetic workup revealing a homozygous valine COMT polymorphism. The temporal relationship between cannabis use and the emergence of psychosis supports a diagnosis of a Cannabis-Induced Psychosis. The persistence of psychosis for four months after cessation of cannabis suggests her ongoing psychosis was due to neurobiological changes, perhaps secondary to persistent cannabis use, but not a direct effect of concomitant drug use. When coupled with cannabis use, individuals with the homozygous valine variant of COMT may have an increased risk of developing psychotic disorders [7]. Given the absence of other significant medical findings that might have contributed to her psychosis, this case implored further exploration between cannabis use and genetic factors like COMT polymorphism in the onset of schizophrenia-like psychosis.

To establish the relation between cannabis use, Val/Val COMT polymorphism, and development of psychosis, there are many variables and confounding factors to consider. The causation should not be assumed without further methodological studies to account for them. We therefore conducted a literature review to determine the likely role of cannabis use and COMT polymorphism in psychosis. We aimed to identify peer-reviewed articles, and searched the reference list of eligible articles to identify additional articles relevant to this study. Screening for eligible articles was conducted independently by five authors. Eligible studies were those that focused on the interactions between COMT polymorphism, cannabis and psychosis. Given the paucity of articles on this topic, all types of studies were considered for analysis including experimental, cohort, case-control, case series, and case reports. Where there were multiple articles reported on the same study population, only the most recent publications were included in our final review. Any disagreement regarding the eligibility of an article was resolved by discussion among the authors. Endnote was used for citation management and de-duplication of references. Relevant data from eligible articles were extracted and entered into a data abstraction form. Table 1 outlines the summary of research on the associations between COMT polymorphisms, cannabis and psychosis.

**Table 1:** Summary of article findings pertaining to COMT Val158Met polymorphisms and cannabis use in relation to psychosis. AoP: age of onset of psychosis, y: years, DUP= duration of untreated psychosis

Article	Sample size, genetic findings, percentage of each polymorphism	Associated findings
Caspi et al. [7]	N= 803 Patient distribution: Val/ Val 25%, Val/Met 50%, Met/Met 25% (in Hardy Weinberg equilibrium)	The relationship between genotype and cannabis exposure was significant. Cannabis use in adolescence was associated with risk of schizophreniform disorder in Val/ Val COMT carriers (OR=10.9, 95% CI= 2.2-54.1), but not in Met/Met carriers (OR=1.1, 95% CI: 0.21-5.4). Psychotic symptoms were reported by age 26 in those with Val/Val genotype (p=0.002),and Val/Met genotype (p=0.001), but not in Met/Met individuals (p=0.55).
Pelayo-Terán et al. [8]	N= 169, (104 diagnosed Schizophrenia) Mean age= 27.4 y Mean age of psychosis onset= 26.17 +/-7.24 y 43 COMT Val/Val (29 diagnosed schizophrenia) 102 COMT Val/Met (67 diagnosed schizophrenia) 24 COMT Met/Met (8 diagnosed schizophrenia)	The link between cannabis use and COMT genotype was significant for both AoP (p=0.024) and DUP (p=0.039). When cannabis was not used, DUP was statistically longer in Val/Val compared to Val/Met and Met/Met. Post hoc analyses revealed no significant difference between COMT genotypes in cannabis users. Cannabis use may lead to earlier onset of psychosis in those with met allele.
Estrada et al. [9]	N= 157, Mean age= 17.01 y 47 COMT Val/Val 77 COMT Val/Met 33 COMT Met/Met	AoP in cannabis consumers: COMT Val/Val (15.46 y) < COMT Val/Met (17.12 y) < COMT Met/Met (18.78 y). Age at first cannabis use correlated with age of onset of first psychiatric disorder. Patients with Val158Met COMT who consumed cannabis at younger ages were hospitalized earlier for schizophrenia spectrum disorders than those with other COMT variants.
Lodhi et al. [10]	N= 169 Mean age= 27.71 y Hardy Weinberg equilibrium: val allele 0.47, met allele 0.53	AoP in those consuming cannabis before age 20: COMT Val/Val (19.37 y) < COMT Val/Met (20.95 y) < COMT Met/Met (21.24 y): Close to significance (p=0.051). Median AoP in males was 20.61 y, and in females was 21.66 y (p=0.0086). Significant analysis showing earlier psychosis in males than females.
Nieman et al. [11]	N= 147 39 COMT Val/Val 65 COMT Val/Met 43 COMT Met/Met	Greater severity of positive symptoms in weekly cannabis users, especially those with Val/Val COMT. Results suggest Val/Val COMT carriers have greater sensitivity to cannabis and thus greater severity of positive symptoms with cannabis use than Val/Met and Met/Met COMT. More negative symptoms in cannabis smokers irrespective of genotype.
Zammit et al. [12]	N = 493 for investigation of interaction between cannabis and COMT in patients with schizophrenia	Found no interaction between cannabis use and Val158Met COMT in subjects with schizophrenia
Ermis et al.[13]	Total N=74, 58 COMT Val/Val(78.4%) 16 COMT Val/Met+Met/Met (21.6%) Schizophrenia with premorbid cannabis use N= 36 32 COMT Val/Val (88.9%) 4 COMT Val/Met+Met/Met (11.1%) Schizophrenia without premorbid cannabis use N= 38 26 COMT Val/Val (68.4%) 12 COMT Val/Met+Met/Met (31.6%)	The rate of schizophrenia with the Val/Val genotype was significantly higher in patients with premorbid cannabis use (88.9%) compared to those without premorbid cannabis use (68.4%). Val/Val genotype increased the risk of schizophrenia 3.69-fold. The mean total PANSS score of the Val/Val genotype group was significantly higher (58.18±1.9) than the scores of the patients with Met allele (50.06±2.85) (p=0.04).
Henquet et al. [14]	N=56 (31 with psychotic disorder, 25 controls) COMTVal158 genotype distribution: 14% (Met/Met), 55% (Val/Met) and 30% (Val/Val) (in Hardy Weinberg equilibrium)	COMT Val158Met: Val allele showed increased hallucinations after cannabis exposure compared to Met/Met genotype. Val/Val genotype most sensitive to effects of cannabis in relation to psychosis.
Henquet et al. [15]	N= 62 (30 with psychosis: n=11 schizophrenia, n=11 schizoaffective, n=8 psychosis not otherwise specified; and 32 controls) Mean age = 27.4 years (SD= 8.7, range= 18-56) Mean score on PANSS was 48.3 (SD=11.5) in patient group and 33.0 (SD=3.0) in the controls COMTVal158 genotype distribution: 26% (Met/Met), 27% (Val/Val), and 47% (Val/Met) (in Hardy Weinberg equilibrium)	Significant difference in the effect of cannabis on psychotic symptoms with greatest effect on Val/Val genotype carriers. Val allele carriers found to be more sensitive to Δ-9-THC induced psychotic experiences, memory and attention impairments compared to Met allele carriers. Δ-9-THC experimental effects were moderated by the COMT Val158Met genotype.
Gutiérrez et al.[16]	N=283 [91 (66 men and 25 women) diagnosed with schizophrenia, 192 (96 men and 96 women) controls] Schizophrenia patients 31 COMT Val/Val (34%) 45 COMT Val/Met (50%) 15 COMT Met/Met (16%) Controls 52 COMT Val/Val (27%) 98 COMT Val/Met (51%) 42 COMT Met/Met (22%)	Val/Val homozygosity was more frequent in the schizophrenia group compared to controls (34% compared to 27%; OR = 1.39; 95% CI, 0.78-2.47). Maximum risk of schizophrenia found in women who regularly consumed cannabis and who were also homozygous for the Val allele. Val/Met heterozygous females who consumed cannabis had an intermediate risk and, Met/Met homozygous females who used cannabis had the least risk (p = 0.152). Findings did not reach statistical significance due to a lack of sampling capacity.

Evidence also suggests that cannabis use in adolescence increases the risk of developing clinically significant psychotic symptoms and schizophrenia later in life [7]. A large proportion of cannabis users, however, do not ever develop these psychotic symptoms [7]. This indicates that genetic susceptibility may be a significant factor in the emergence of cannabis-induced psychosis. Caspi et al. conducted a longitudinal study of a representative cohort followed from birth to adulthood [7]. The results demonstrated that carriers of the COMT Val158Met allele (representing a substitution of valine by methionine) had an increased risk of developing psychosis related symptoms in adulthood if they had used cannabis in adolescence. The study emphasizes that "the presence of such a gene-environment interaction is indicated by the finding that the association between cannabis and psychosis outcomes is most marked in subjects with an established vulnerability to psychosis” [7]. This literature review further highlights a notable difference in age of onset of psychosis in early cannabis users with COMT polymorphisms, with a tendency for Val/Val homozygotes to manifest symptoms at younger ages. It is proposed that cannabis use may alter onset based on COMT genotypic makeup. Although cannabis use may predispose individuals to psychosis irrespective of genetic makeup, our findings support an association between early cannabis use and early-onset psychosis in Val/Val COMT individuals.

Bosia et al. [5] substantiated a significant interaction between the COMT mutation and skills such as verbal frequency and speed of processing. Their results suggested a negative effect on both skills in patients who use cannabis with the Val/Val polymorphism compared to patients who use cannabis with either Met-COMT variant. In addition, a 2008 study conducted by Pelayo-Terán et al. demonstrated a considerable correlation between valine allele COMT carriers, an earlier age of onset of psychotic symptoms, and an increase in severity of negative symptoms [8]. The diagnosis of schizophrenia was found to be prevalent in patients with a Val/Val or Val/Met genotype, and patients who had a homozygous valine genotype experienced more severe negative symptoms. This was in concordance with previous studies that had shown the homozygous valine genotype to have a higher degradation of prefrontal dopamine by the COMT enzyme, resulting in decreased cognitive function and brain abnormalities [12]. These findings suggest that the presence of the valine allele promotes reduced prefrontal dopaminergic transmission in patients experiencing an initial episode of psychosis, which could cause an early onset of psychotic symptoms, development of schizophrenia and a greater severity of negative symptoms. Our case correlates with and further supports these findings and suggestions. 

A case-only study conducted with a retrospective assessment of cannabis use further evaluated the relationship between cannabis and COMT variation in 158 individuals with schizophrenia [6]. This was one of the largest sample sizes of patients with schizophrenia that looked at the interaction between cannabis, COMT, and schizophrenia. The individuals in this study were sampled from two different geographic locations and their genetic profiles were compared. The results were consistent between the two groups and revealed that patients with schizophrenia who were homozygous for the Met allele at rs4680 were more likely to use cannabis. Although these results conflict with the previously described study [7], it further corroborates a relationship between COMT polymorphisms, cannabis use, and schizophrenia. Another study found a 10-fold increased risk in developing schizophreniform disorder by age 26 in cannabis users who carried the rs4680 COMT variation [17]. 

In a cohort study involving The Avon Longitudinal Study of Parents and Children (ALSPAC) [18], the connection between cannabis use and ensuing psychotic disorders was established, but there was no proof of association between COMT variants at rs4680, cannabis use, and the susceptibility of developing psychosis using a multiplicative model. Although the study examined six single nucleotide polymorphisms (SNPs) within COMT at rs4680, they failed to establish any statistically significant relationship even after adjusting for probable confounders. Furthermore, a retrospective study by De Sousa et al. [19] established an earlier age of onset of schizophrenia in male subjects who used cannabis compared to female cannabis users with schizophrenia, but failed to substantiate an association between cannabis use, COMT genotypes and schizophrenia [19].

Based on the literature review, we found an inconsistent relationship between cannabis use and COMT polymorphisms in individuals with schizophrenia or schizophrenia-like symptoms. Studies that have established a significant association between COMT rs4680, cannabis use and schizophrenia include those by Capsi et al. [7], Pelayo-Terán et al. [8], Estrada et al. [9], Lodhi et al. [10], Nieman et al. [11], Ermis et al. [13], and Henquet et al. [14]. The ALSPAC cohort study [18] along with De Sousa et al. [19], and Zammit et al. [12] failed to establish this association across the studied COMT SNPs, including the commonly implicated rs4680 COMT variation [18]. Despite these discrepancies, we found the majority of the studies included here suggest that early cannabis use in individuals with homozygous valine COMT polymorphism is associated with an increased risk of developing positive and negative symptoms of schizophrenia later in life. The age at first cannabis use was found to be positively correlated with the age of onset of the first psychotic episode. These findings were supported by our patient with Val/Val COMT polymorphism, who began using cannabis at 13 years of age, and experienced early-onset symptoms of schizophrenia at age 24. Age of onset of psychosis in our patient falls in the range suggested by Desousa et al. [19] and Pelayo-Terán et al. [8], where the female cannabis users had the psychosis onset at the mean age of 22.5 y (SD 6.5), and 26.17 y (SD 7.24), respectively. Our findings provide additional evidence to support the influence of environmental factors in the development of schizophrenia and the vulnerability of certain genes to environmental substances such as cannabis. According to previous research, the Val-COMT allele is associated with increased hallucinations after cannabis exposure [9]. Although our patient developed other positive symptoms, she did not experience any hallucinations. She presented predominantly with the negative symptoms such as anhedonia, lack of motivation, poverty of speech, decreased speech frequency, and increased speech latency and verbal processing. The abundance of negative symptoms demonstrated in this case is also evident in previous research [5,8]. Therefore, our case supports literature that suggests an increased risk of developing symptoms of schizophrenia in cannabis users with Val/Val polymorphism compared to either Met-COMT variants, as well as an association between early cannabis use and psychosis in Val/Val COMT individuals. 

This case report adds to the limited existing literature on this topic and combines the findings of various studies to support a possible relationship between cannabis use, age of onset of psychosis, Val/Val COMT polymorphism, and development of schizophrenia. We recommend further research to investigate the details of the association between cannabis use and schizophrenia in genetically predisposed individuals. The future studies may include extensive examination of prodromal symptoms, gender variations in relation to age of presentation of first psychotic episode in cannabis users, and further exploration and focus on potential linkages to COMT, particularly those with Val/Val genotype, and neurobiological mechanisms implicated by said relationship.

## Conclusions

Schizophrenia is a mental disorder with complex etiology, involving the combination of genetic, environmental, and neurochemical alterations. Cannabis users with homozygous valine COMT polymorphism are at increased risk for developing schizophrenia. Further studies are needed to understand the detailed underlying mechanisms in the interplay of COMT variations, cannabis use, and schizophrenia development.
